# Des Maladies Mentales

**Published:** 1839-01-01

**Authors:** 


**Des Maladies Mentales, considerées sous les Rapports Medical, Hygiénique, et Médico-Légal.** Par *E. Esquirol*, &c. &c. &c. Accompagnées de 27 Planches Gravés. Deux Tomes. Paris, chez J. B. Bailliere, 1838.

**On Mental Disorders, &c.** By *E. Esquirol.* Two Volumes 8vo., with 27 Engravings; Paris, 1838.

[Continued from No. 58.]

In our last number we presented a sketch of M. Esquirol's account of the symptoms and causes of insanity. We take up that account at the progress and treatment of the malady.

M. Esquirol observes that almost all the insane whom he has seen presented some disturbance in their functions for a longer or shorter period previously to the attack. The majority had had acute cerebral inflammations, convulsions, headaches, colic, cramp, constipation, or menstrual irregularities. Many too had displayed mental peculiarities of some description —great intellectual activity—violent passions—whimsical ideas, affections, or actions; and so forth.

M. Esquirol justly remarks that the first palpable outbreak of insanity has generally been preceded by indications of it which escaped attention. The insane frequently struggle against their hallucinations, long before they become apparent to the world. The habits, tastes, sentiments of the insane, have been changed before the actual outbreak occurs. That outbreak is frequently considered the commencement and the source of the disorder, when, in point of fact, it is but the denouement of what has been long forming. A man gave himself up to extravagant speculations which failed. He went mad. In point of fact, he was mad before his failures. A man became outrageously evangelical, assisted at a meeting from which he issued in a state of terror, believing that he must be damned. It was not the sermon that turned his head—it was turned already. A young nobleman set off on his travels for several years, eight days before his wife was to be confined. Some annoyances happened en route, and in six months he was stark mad. But was not the very journey the first act of madness? M., aged 64, of a nervous temperament, had always led an irreproachable life. He began to go frequently from home, under the pretence of taking walks. His wife grew uneasy, and directed his valet-de-chambre to watch him. The servant traced him to a brothel of the lowest sort. On the slightest hint of the discovery, he flew into a paroxysm of fury, which, in five days, ended in dementia.

We consider these remarks, and these illustrations, fraught with practical value. They bear on a point which may be said to be at present under discussion in this country—whether patients are too soon and too often, or too late and too seldom subjected to restraint as lunatics. Our own opinions have been often expressed—are expressed in another part of this Journal.* We are glad to perceive that we have the sanction of Esquirol's

* See the Periscope of this number.

immense experience in the view which we have taken. It is scarcely possible, in this country at all events, for a sane man to be incarcerated as a lunatic; but it constantly happens that lunatics are allowed to go abroad as sane men.

Insanity may be continued, remittent or intermittent.

Continued insanity has regular phases, at least acute and accidental insanity has them. Those phases or epochs are three;—a first acute, with concomitant symptoms—a second chronic, unmarked in general by any symptoms, save the illusion—a third, that of the decline and cure. These successive periods are not observed in idiotcy nor in dementia.

Remittent insanity presents remarkable anomalies both in its character, and in the duration of the remission. In some cases, the remission is only the transition of one form of mania to another. Thus an insane person passes three months in the condition of lypomania, the next three in that of mania, and four, perhaps, or thereabouts, in that of dementia. Thus these forms may appear in succession, sometimes with regularity, sometimes with very great variations. A lady, aged 52, is one year lypomaniacal, and one year maniacal and hysterical.

In other cases, the remission exhibits only a diminution of the symptoms or the kind of madness. Thus some maniacs are only violent at certain times of the day, or on certain days, or at certain seasons. With other madmen, melancholia is only overwhelming at intervals, the insanity being usually of some definite kind, combined with the gayer passions. The seasons, menstruation bring on the same symptoms, the same illusion, the same excitement, and the same depression.

Intermittent madness is quotidian, tertian, quartan, monthly, annual, or with intervals of several years. The intermission may be regular or irregular. If the former, the same season, the same causes, resuscitate a disease with the same character, the same crises, the same duration as before. Most commonly the paroxysms recur after irregular intervals, are provoked by new causes, do not affect the same form. It may explode at once, or be preceded by symptoms similar to those which ushered in the first attack.

Besides the conversion of one form of insanity into another, several forms may be combined; melancholia, for example, with mania—dementia with mania and monomania.

Insanity is often complicated with cerebral lesions, such as chronic inflammation of the membranes, paralysis, convulsions, epilepsy, hysteria—or with affections of the lungs, the heart, the intestines, or the skin. These latter affections may have preceded the complaint, and ceased when it appeared, or they may concur or alternate with it.

Intercurrent or epidemic maladies may attack the insane, and exert a greater or a minor influence on their mental alteration.

M. Esquirol, who certainly strikes us as in many respects a Hippocratist, leans to the doctrine of crises in insanity. He ranks them as either physical or moral. They are only observable in monomania, melancholia, mania, or acute dementia.

Insanity may terminate by resolution. The restoration of the natural complexion and expression of the features, bodily lassitude, sleep, the restoration of the secretions of the skin and of the excretions, the re-establishment of healthy moral feelings:—such are the precursors of recovery. This is complete if reason returns, if habitual evacuations are re-established, and if old habits and the natural character are resumed. But if the bodily functions are restored, and the mental alienation persists, or the "moral sensibility" is not re-established in the same proportion with those functions, the mania or the monomania will become chronic, and degenerate into dementia.

Sometimes insanity terminates in obesity, the former declining as the latter augments. But obesity combined with a persistence of the alienation is a sign of dementia. On the other hand, extreme emaciation is also a termination of insanity, the patient only regaining his reason "when he has absolutely knocked at the gates of death." M. Esquirol is quite convinced that this emaciation is a critical termination of insanity, not a mere effect of it. He mentions a case in corroboration of his view.

*Case.* Madame ——, aged 51, had had several attacks of mania, as a consequence of severe afflictions, each attack ceasing so soon as she became extremely emaciated. An intermission occurred of two years' duration, when she grew extremely fat, and appeared to have attained the very pitch of good health. This was the signal of a sudden outbreak of insanity, which lasted for some months, diminished when emaciation commenced, and ceased when this had gone to some extent.

M. Esquirol assures us that he has often witnessed cases of a similar description.

He cites authorities to prove that insanity often terminates in fever—and in hæmorrhoids. The first menstrual flux is sometimes critical—the cessation of the menses decidedly is so. M. Esquirol has seen several women recover their reason on the cessation of the catamenia. He has seen too the re-establishment of the menses put an end to insanity, as uterine hæmorrhages, leucorrhœa, gonorrhœa, have done. Coitus and seminal discharge have proved critical; so have gestation and lactation. But M. Esquirol is of opinion that marriage has been too hastily recommended for insanity. It is not nearly so often advantageous as is supposed, and sometimes it aggravates the evil.

Cutaneous affections assume some importance in relation to insanity, from the circumstance that their suppression causes insanity, and that the insane are very prone to them. M. Esquirol agrees with Hippocrates in believing that the itch proves critical. He attempted to communicate it to a soldier, who had fallen into dementia and paralysis after the repression of scabies. But he neither gave the soldier the itch nor cured him. Boils followed by a pretty copious discharge often terminate insanity; but enormous atonic formations of matter never have that favourable effect. When suppressed ulcers cause the disorder, their re-establishment cures it.

Perfect and Pinel relate the cure of insanity after swelling of the parotid. A woman, aged 40, at the Salpetrière, who had become maniacal from alarm at a thunder-storm, lost her complaint on the occurrence of an enormous enlargement of the submaxillary glands; she fell into a profound state of stupor, which subsided with the subsidence of the glandular swelling.

Lafontaine read to the Society of Goettingen, the history of an insane person, who, after the lapse of several years, was cured by the extirpation of a cancer in the breast.

Salivation is a very common occurrence with the insane. Many seem on the point of spitting, yet no saliva appears. Salivation may be critical.

It is the same with the flow of tears. Cutaneous transpiration is a much more frequent termination of insanity than is commonly supposed. It is on this account that the season of Spring, and that warm-baths tend towards the production of a cure.

Vomiting of mucous matters of a yellow or a dark colour, and alvine dejections of the same character, often terminate insanity, especially melancholia. The expulsion of worms has been attended with the same result. But it does not therefore follow, as M. Esquirol rightly observes, that insanity depends on gastro-intestinal irritation. Many diseases whose seat is remote from the abdomen, are relieved by abdominal evacuations. This is no doubt true, yet abdominal irritation may prove the exciting cause of insanity, in persons predisposed to it.

One form of insanity may run into another, and thus the latter may prove critical to its predecessor. In some individuals, insanity is succeeded by hypochondriasis, or hysteria. M. Esquirol has never seen insanity cease on the supervention of epilepsy. Epileptiform convulsions announce encephalic lesion, and bode the death of the patient.

M. Esquirol examines the question, whether strong moral impressions may not cure insanity. He mentions several cases. We shall select one or two.

*Case.* A young lady fell into melancholia because she was disappointed in marrying her lover. She refused all nourishment, and lapsed into a state of marasmus. After some months, her lover appeared before her with the assurance that they would be shortly married. She was cured.

*Case* 2. A lunatic refused all nutriment, because honour forbad him to eat. After ineffectual attempts for some days to conquer his resolution, an ordonnance was taken to him, with the forged signature of "Napoleon," commanding him to eat; and absolving him from all imputations on his honour if he did so. The lunatic read the order, hesitated for some hours, obeyed, and was saved.

Some further cases and several reflections end in the conclusion, on the part of M. Esquirol, that moral treatment is of great efficacy in cases of insanity—a conclusion reasonable in itself, and the foundation of the great improvements that have been effected in the management of lunatics in this country.

M. Esquirol next examines the proportions of cures and deaths in the disorder. We shall advert to this point and to the data on which it rests, more particularly in an article on the statistics of lunacy. We may merely state generally at present, *that* the number perfectly cured is about one-third of the affected; *that*, the proportion ranges under the influence of circumstances, locality, and so forth, from one-fourth to one-half; *that* the cures are more numerous in France than in England, and least numerous in Germany and Prussia. M. Esquirol insists on this point for the double purpose of repressing the ostentation of the English in reference to their treatment of insanity, as well as for that of giving a gentle rub to his countrymen, who think that whatever is foreign is best. This is certainly a new character for our French neighbours to appear in. We never before heard that accusation urged against them. M. Esquirol would stare could he be transplanted to this country, and observe how prevalent the cacoethes that he mentions is here.

But the period occupied in obtaining the cure is as important a consideration as the cure itself.

M. Esquirol has constantly observed that the mania reaches its height, and begins to decline during the first month. It is during the first month, compared with all succeeding ones, that the greatest number of cures are obtained.

The mean duration of insanity has been fixed by Pinel at between five and six months. Mr. Tuke, in his report of the Retreat at York, has extended the period considerably, and M. Esquirol agrees with the latter. His own observations, founded on admissions into the Salpetrière, lead him to conclude—*that,* the greatest number of cures are obtained in the two first years;—*that* the mean period of cure is rather under a year;—*that* after the third year, the chances are 30 to 1 against cure.

But M. Esquirol warns us against despairing of a case of insanity, however ancient, and however unpromising. Without referring to cases recorded by others, he has himself seen several instances of recovery after a long period. A young lady had been for ten years in a state of dementia, attended with suppression of the catamenia. One day she jumped up and ran to embrace her mother, crying out—" Mamma I am quite well." Her menses had begun to flow spontaneously, and her reason was re-established. M. Esquirol has twice seen insanity disappear at the turn of life, in women who had been insane from their earliest youth. But still it must be owned that these fortunate cases are rare.

The greater number of recoveries are observed in Spring and Autumn.

The most favourable age for recovery is between twenty and thirty. After fifty a cure is rare.

Mania and monomania are more curable than melancholia. Chronic dementia is seldom cured. Idiotcy and dementia senilis are incurable.

Some lunatics are curable only to a certain point. They are so susceptible to mental impressions, that the slightest causes occasion a relapse. Seclusion in a retreat alone preserves their reason. The mental powers of others are so shaken, that they are unable to resume their parts in the world, although they are not irrational. Recoveries of this kind bear the proportion of about one-twentieth to the rest.

But it not unfrequently happens that individuals considered cured by their friends and even by their medical attendant, have still some lurking remains of their malady. M. Esquirol mentions several cases of this sort. We may quote one.

*Case.* A lady cured apparently of an attack of suicidal melancholia with paroxysms of mania, passed a month at Paris, in order to recruit both mind and body, and then returned to her family. Every one felt convinced of her perfect restoration to reason. A year afterwards her husband was seized with apoplexy. The shock, and the necessity for exertion, in order to prevent ill consequences to her children, dispelled insane ideas which had occupied her mind during a whole year of apparent restoration to reason.

It is a general opinion that relapses after recovery from insanity are very common. M. Esquirol thinks their frequency over-rated. He estimates them at one tenth of the recoveries. Relapses are more frequent amongst the poor than the rich, for the obvious reason that the former can less effectually guard against the exciting and indeed predisposing causes of the disorder. Even the relapses that do occur, might, in M. Esquirol's opinion be, in a great measure, avoided.

Our author agrees in the main with those who believe that insanity tends to shorten life, although there are many instances of longevity on the part of lunatics.

The mortality will seem greater in establishments in which lunatics are received indiscriminately, than in such as select the cases most likely to be cured. This, according to M. Esquirol, explains the comparatively low mortality in the hospitals of London and York, and the comparatively high rate at the Salpetrière, the Bicêtre, and the Charenton.

The mortality is greatest in Autumn and Winter. In this latter season the causes of ill-health and disease are most rife, and lunatics, like other people, must naturally suffer from them.

While the age from twenty-five to thirty-five is that which is most favourable for the production of insanity, from thirty to forty is the period in which the mortality is greatest. This is particularly the case with men. With women the deaths are most numerous between the ages of forty and fifty.

M. Esquirol's estimate of the mortality is as follows:—

Taking insanity in general, he ranks it at from 1 in 6 to 1 in 8.
In Mania, it is 1 in 25.
In Monomania, 1 in 16.
In Melancholia, 1 in 12.
In Dementia, 1 in 3.

Idiots are incurable, and seldom live beyond thirty or forty years.

Acute accidental mania is seldom fatal. Simple melancholia, even when characterised by the inclination to suicide, is only mortal when produced by an organic lesion, or when complicated with severe disease. Dementia being the final term of all the forms of mental alienation, is the most frequently fatal. It is often combined with paralysis, and it is this circumstance which renders the mortality at the Bicêtre and Salpetrière so high.

*Causes of Death.*—The principal alterations which cut off the insane are meningitis, cerebral fever, apoplexy, lesions of the brain, thorax, or abdomen. Two-eighths of the fatal terminations are laid by our author to the door of cerebral affections, exclusive of epilepsy and paralysis—two-eighths to diseases of the thorax—three-eighths to diseases of the abdomen. Notwithstanding that Greding and Monro affirm that marasmus and dropsy of the chest destroy the greater number of lunatics, the examination of the bodies of six hundred patients has not led M. Esquirol to the same conclusion. He has found the thoracic less numerous than the abdominal lesions.

Melancholia often ends by low nervous fever. Sometimes the patients obstinately refuse to quit the bed—sometimes they crouch upon the ground. Some obstinately refuse all nourishment—others eat with frightful voracity. They emaciate, fall into extreme debility, are attacked with fever marked by nocturnal exacerbations, and diarrhœa often comes to accelerate their fate.

The phthisis which complicates mania, and more particularly melancholia, has been noticed by many observers. M. Esquirol has frequently seen it precede or appear along with it. The phthisis, in these cases, is very likely to be overlooked. The mental alienation augments even to the end. It is not the exertions of the lung in their vociferations that produce the phthisis, for this is a more frequent attendant on melancholia than on furious mania. Sometimes insanity alternates with phthisis, and the latter subsides during the paroxysm of the former.

Scurvy is another very common concomitant of insanity. It is no doubt due to bad management. We willingly believe that at present it is rarely seen in this country, and that it will ultimately disappear in all. Insanity there must always be—scurvy need not exist.

Half of the insane who die, especially the monomaniacs, are paralytic. Apoplexy is another disease which often destroys the insane. Of two hundred and thirty-seven individuals, thirty-seven died apoplectic.

M. Esquirol gives the following table of the diseases which proved fatal in 277 patients.

| | |
|---|---|
| Adynamic Fever | 32 |
| Ataxic Fever | 14 |
| Cerebral Fever | 28 |
| Low Nervous Fever | 25 |
| Pleurisy | 12 |
| Phthisis | 28 |
| Latent Peritonitis | 13 |
| Colliquative Diarrhœa, Scorbutus | 38 |
| Hydro-pericarditis | 11 |
| Scirrhus of the Pylorus | 4 |
| Organic Lesions of the Liver | 35 |
| Apoplexy | 33 |
| Epilepsy | 4 |
| | 277 |

*On what Cerebral Alterations does Insanity depend?*—This important question is almost answered by an admission of ignorance on the part of M. Esquirol. He offers, however, a general summary of the results hitherto obtained in this inquiry.

1. Malformations of the cranium are only observed in imbeciles, idiots, and cretins.

2. Organic lesions of the encephalon or its envelopes have only been observed in those whose insanity was combined with paralysis, convulsions, epilepsy. The lesions belonged to the complication, not to the insanity.

3. The sanguineous or serous effusions—the injections or infiltrations—the thickening of the membranes—the ramollissement, induration, tumors of the encephalon, &c.—all these alterations indicate the causes or the effects of insanity, or rather the effects of its complication with the malady which proved fatal to the patient.

4. The alterations of the thorax, abdomen, pelvic cavity, are evidently independent of insanity. Yet they may point out the organ whose alterations first disturbed the brain.

5. All the organic lesions found in the insane have been found in those who have never exhibited insanity.

6. In many instances, examination of the body after death has led to the discovery of no alteration, although the insanity had existed for years.

7. Pathological anatomy shews us each part of the encephalon altered, or destroyed, without the occurrence of insanity.

8. From all these data we may conclude, *that* the immediate cause of insanity is beyond our means of investigation; *that* the disorder depends on some unknown modification of the brain; and *that* it does not always originate in the brain, but in some of the seats of sensibility placed in the various regions of the body. This may seem discouraging, but if true, should be well known. Perhaps, as M. Esquirol observes, an exact acquaintance with the immediate cause of insanity would not help us much in the treatment of it. Probably, if we were exactly acquainted with the subtle condition of the nervous system which produces pain, we should not be able to lull it one whit better than we do.

*Prognosis of Insanity.*—Imbecility and idiotcy are never cured.

Monomania and melancholia, when recent, accidental, and independent of organic lesion, are curable.

Mania is more curable than monomania and melancholia.

Acute dementia is sometimes cured—chronic dementia is rarely cured—and senile dementia never.

Hereditary insanity is curable, but relapses are more probable than in accidental insanity.

Chronic insanity is with difficulty cured, especially after the second year; the difficulty is augmented in proportion to the length of time the causes have operated prior to the occurrence of the malady.

However long the insanity may have existed, recovery may take place while palpable derangements of the corporeal functions exist.

If the moral causes have been sudden in their operation, the prognosis is more favourable, than if they have acted gradually.

If insanity has been produced by excess of study, the prognosis is unfavourable, especially when errors of regimen have been combined with the over-exertion of the mind.

Insanity dependent on religious excitement, or on pride, is seldom cured.

Insanity kept up by hallucinations is very difficult of removal.

When the insane are well aware of their condition, yet are not promptly cured, the prognosis is unfavourable.

When the insane recover their bodily health, yet evince no progress towards mental restoration, we must not be sanguine of their recovery.

When the senses of the insane are so enfeebled that they can look upon the sun without uneasiness, and have lost smell, and taste, &c., they are incurable.

Insanity is incurable when the consequence of scurvy, or epilepsy. Its complication with these diseases and with paralysis is invariably fatal.

Such is the summary of data for prognosis, with which M. Esquirol presents us.

We pass to the *treatment.*

The leading objects are to calm their passions—to remove the mental infirmity or aberration—to remove the physical disturbance. Each case must be studied—the causes of the disease investigated—the character of the patient determined. There is no specific treatment for insanity. As the causes of the malady are moral or physical, the means of treatment must be physical and moral too.

*Separation of the Insane.*—M. Esquirol adds his opinion to the weight of sentiment in favour of the separation of the insane from their friends. He remarks that strangers who become insane in Paris are more easily cured than the Parisians, because they are more completely separated from old scenes, faces, and associations of ideas. This is now so generally admitted that it is useless to argue on it.

It is certainly singular, that the insane are usually most suspicious and conceive the greatest aversion for those who were previously dearest to them. Before strangers they will often be calm and rational, while they are evincing the very reverse of calmness and of reason to their relatives and friends.

As a general rule, M. Esquirol prefers confinement of the insane in an establishment to confinement in private. In point of fact, he has found that they are more easily cured in the former than in the latter situation. We need not enter on the why or wherefore. No doubt, it is the variety, the withdrawal of the insane from himself and from his own sensations, that facilitates recovery in the public establishment.

But M. Esquirol admits that there are instances, in which separation of the insane from friends is not advisable.

An establishment should have but one Director, and he should be a medical man. If the directors are more numerous, authority is divided, and that obedience so necessary for the advantage of the insane is apt to be interrupted.

It is not easy to determine the period at which confinement should terminate. Great judgment and tact are requisite on the part of the medical man. If separation has been maintained for some time without effect, M. Esquirol advises that the relatives or friends should be permitted to visit the patient. The visits should be sudden and unexpected, in order that the effect on the patient may be considerable. But during and after convalescence, great prudence should be exercised in permitting and in regulating the visits of friends. As a general rule it is better to sin on the side of too long than on that of too short a confinement.

At the commencement of insanity, the mental alienation is difficult to be distinguished from, and likely to be confounded with, febrile delirium. Wherever there is any doubt, it is better to wait and allow the affection to develop itself fully, before any measure like confinement is determined on.

In dementia and idiotcy, confinement is only necessary for the prevention of accidents, and for the safety of the individual. Maniacs and monomaniacs must necessarily be confined. Poor lunatics must, for obvious reasons, be so too. But confinement does not suit some cases of melancholia.

M. Esquirol gives one caution—not to enter on long arguments with the insane. They should be addressed in accents of reason and kindness, but their insanity will assuredly not be put out by syllogisms.

*Music.*—The ancients vaunted its effects. M. Esquirol has employed it. He has very rarely seen patients cured by it. Yet it calms and composes the mind of the lunatic. M. Esquirol, however, has seen it produce fury—in one case, because the madman swore that all the notes were false; in another, because he was incensed at the idea of any one's amusing himself in the vicinity of such an unfortunate as himself.

*The Theatre.*—Both reason and experience contribute to prove, that in madness "the play is" *not* "the thing." While theatrical representations were permitted at the Charenton, one seldom occurred without some outbreak of delirium or relapse. In three instances, to which he refers, M. Esquirol selected both the patients to take, and the theatrical representation they were to witness. One of the patients was a young convalescent whom he escorted to the opera-comique. He saw nothing but his wife flirting with men. Another, after a quarter of an hour, felt his head hot—"let us go out," said he, "or I shall relapse." A third was a young lady whom he accompanied to the opera. Seeing the actors armed with swords, she thought that they were going to fight, and it was necessary to take her out to prevent mischievous consequences.

*Travelling.*—M. Esquirol speaks favourably, on the whole, of this remedy. He has always observed that the insane are relieved after a long journey, especially if they have visited foreign countries, and have had their attention awakened by new scenes. Convalescents too find benefit from travelling. It occupies their thoughts, and can form the subject of their conversation to their friends.

M. Esquirol next proceeds to physical treatment; the preceding remedies have been of the nature of moral ones.

The abode of the insane should be chosen in reference to the general and well-understood conditions of salubrity. It is a great blunder to imagine that the insane do not feel atmospheric vicissitudes. Their constitutions are enfeebled, and they *do* feel them sensibly.

In acute insanity the spot should be open, but the residence should be retired. In chronic insanity the same degree of retirement is unnecessary. In melancholia the retreat should be pleasantly and, if possible, picturesquely situated. If the insanity has appeared in a hot climate, the return to a cold one may reasonably be expected to prove beneficial.

*Dress.*—The insane, especially melancholics, should be warmly clad; frictions of the skin and the use of flannel are of service. In sleeping the bed-clothes should not be too cumbrous, and the head should be, in general, bare.

*Diet.*—On this head we perceive nothing to extract, save a warning of M. Esquirol's against the custom pursued in some hospitals, of distributing, in the morning, the food for the day—a custom so manifestly absurd and objectionable that it is unnecessary to do more than denounce it.

The majority of maniacs and of monomaniacs are devoured with thirst. Appropriate drinks should be allowed them in sufficient quantities.

*Gardening and Farming.* M. Esquirol speaks in high terms of this species of occupation. There can be no question that experience has decided generally in its favour.

At the Salpetrière, the females are employed in a systematic manner. There is a large room in which some are occupied in sewing, others in knitting. Some perform the necessary domestic offices of the establishment —others cultivate the garden.

It is difficult to occupy individuals of the upper classes in this manner. This interferes with their recovery, and goes far to neutralise the advantages they possess in other respects.

*Removal of the Cause of the Insanity, if possible.*—M. Esquirol raises his voice against empirical treatment, that is, against any *one* mode of treating insanity. In this, as in most other disorders, the physician should ascertain if possible the physical or moral cause of the malady, and, if possible, remove it: and his treatment should be also regulated by the nature of the existing symptoms.

If there are excitement and plethora, the antiphlogistic treatment and regimen should be adopted. Almost always, at the expiration of 8, 15, 21, or 30 days, an intermission or remission takes place, and then, combined of course with appropriate moral management, the particular indications of the case should be investigated and acted on. Thus, says M. Esquirol, the patient has been long subject to hæmorrhoidal discharges, which have ceased to flow; the physician re-establishes, or endeavours to re-establish them. Or an ulcer has healed prior to the occurrence of the monomania; the ulcer must, if possible, be re-opened. It is the disposition, on the part of M. Esquirol, to dwell on circumstances of this description, circumstances which, though not unimportant, are erected by him into too great importance, that leads us to deem him a decided Hippocratist. His leaning to the doctrine of critical days, indeed the whole tenor of his observations, would induce us to place him in the category of the followers of the médecine expectante.

But suppose that measures of this description have proved unavailing. The time is come for "empirical remedies."

*Water.*—This has been applied in all varieties of manner.

M. Esquirol thinks that tepid baths, of the temperature of 20° to 25°, (we presume of the centigrade scale,) are most useful. They may be prolonged for several hours in the cases of thin, nervous, and irritable subjects. If there is much determination of blood to the head, bladders filled with cold water may be applied to the head at the time that the rest of the patient's body is kept immersed in the bath.

The cold bath is adapted for young and robust subjects who suffer with heat. Of the warm bath M. Esquirol speaks hesitatingly. He does not appear to have had much experience with it.

The plunging bath and cold affusion are of service to patients who are enfeebled, particularly by masturbation, or by long-continued mental annoyances. The bath of surprise has never been made use of by M. Esquirol, but he is acquainted with fatal results from it. He thinks it would be quite as rational to prescribe pitching the madman out of a three pair of stairs window, because one or two have been cured after a fall on the head.

The douche is mainly useful in young, strong patients, affected with cephalalgia. It ought never to be intrusted to servants, nor administered shortly after a meal, nor ought it to be continued for more than a few minutes. The primæ viæ should be cleared out prior to its use.. It is not totally exempt from hazard.

Ice to the head calms cephalalgia and furor, especially at the commencement of the attack, and when combined with immersion of the feet in hot water, or in a mustard poultice.

Pediluvia are of service. They may be composed of hot water with salt, sal ammoniac, mustard, &c. The water should not be too hot at first. If it is, the kind of shock occasioned reacts upon the cerebrum. The water should be made gradually hotter after the immersion of the feet in it.

Lavements, the douche by the rectum, and copious potations of cold water by the mouth, have been recommended and employed. M. Esquirol seems to speak seriously of the benefits of drinking cold water freely in cases where there is a tendency to suicide.

*Evacuants.*—M. Esquirol responds to the general opinion in favour of emetics. Yet he does not think that remedies of the class of evacuants are adapted for all cases. He is of opinion, and no one can object to it, that the particular purgative should be adapted to the case. He remarks that some patients refuse medicine, protesting that their bodily health is very good. A very good plan is to give such patients, without their knowledge, some substance which will disorder their stomach or bowels, and by rendering them uneasy respecting their health, will conquer their indisposition to physic.

M. Esquirol strongly reprobates the abuse of bleeding—an abuse which has been productive of much mischief to the insane. He has seen a madman bled thirteen times in forty-eight hours. Yet moderate bleeding is occasionally both rational and serviceable, and the local abstraction of blood by leeches, or by cupping, is, in cases of cerebral determination, of undoubted benefit.

*Tonics and Antispasmodics.*—Of great use in particular cases, in which there are indications for them.

*Narcotics.*—M. Esquirol reprobates them.

*Counter-Irritation.*—Not serviceable in the majority of cases, in which it only torments.

*Electricity and Galvanism.*—M. Esquirol has only in one or two cases seen the former of benefit.

*The Circular Swing.*—It is universally abandoned.

We shall return to M. Esquirol's work, and select particular subjects for notice. Our readers will be put in possession of his vast experience on one of the most important of human maladies.

